# Genomic epidemiology of *Acinetobacter baumannii* goes global

**DOI:** 10.1128/mbio.02520-23

**Published:** 2023-11-01

**Authors:** Santiago Castillo-Ramírez

**Affiliations:** 1 Programa de Genómica Evolutiva, Centro de Ciencias Genómicas, Universidad Nacional Autónoma de México, Cuernavaca, México; Institut Pasteur, Paris, France

**Keywords:** genomic epidemiology, *Acinetobacter baumannii*, antibiotic resistance, international clones, carbapenem resistance

## Abstract

*Acinetobacter baumannii* is a major public health concern, for which many genomic epidemiology studies have been conducted in the last decade. However, the vast majority of these are local studies focusing on hospitals from one or a few countries. Proper global genomic epidemiology studies are needed if we are to understand the worldwide dissemination of *A. baumannii* clones. In this regard, a recent study published in mBio is a good step forward. Müller et al. (mBio e2260-23, 2023, https://doi.org/10.1128/mbio.02260-23) sequenced the genomes of 313 carbapenem-resistant *A. baumannii* isolates from over 100 hospitals in almost 50 countries from Africa, Asia, Europe, and The Americas. With this data set the authors provide an updated view of the global distribution of the major international clones and their carbapenemase genes. Future global genomic epidemiology studies can be enhanced by considering not only human but also non-human isolates, and by considering isolates despite their antibiotic resistance profile.

## COMMENTARY

*Acinetobacter baumannii* is a very important nosocomial pathogen. It frequently causes diverse types of infections in critically ill patients in many hospitals all over the world. In particular, carbapenem-resistance *A. baumannii* (CRAB) is very worrisome, as some of these isolates have developed resistance to last-resort antibiotics. As a matter of fact, the World Health Organization ranks CRAB as a critical (priority 1) bacterium for which new antibiotics are urgently needed (1). Considering this, molecular epidemiology is playing an unprecedented role in understanding the spread of clones, their virulence, and antibiotic resistance. Like with many other pathogens, whole-genome sequencing (WGS) has been pivotal in studying this pathogen’s dissemination and characterizing its genetic basis for virulence and resistance (2). In recent years, WGS has become the definitive strategy for conducting molecular epidemiology of this superbug, either using (core) genome phylogenies (3, 4) or core genome multilocus sequence typing (MLST) (5, 6); not least because the typical 7-loci MLST, the goal standard to carry out molecular epidemiology in many bacterial pathogens, is prone to flawed genotyping for *A. baumannii* due to its highly dynamic genome (4).

Genomic epidemiology studies have been carried out to characterize the diversity of clones circulating within and between hospitals in several countries in different regions of the world. For instance, recent studies have analyzed the clones disseminated in hospitals in cities from Mexico (7), Brazil (8), or Egypt (9). The same applies to many other countries in the world. Local genomic epidemiological studies about this superbug have been increasing in the last decade. Typically, these studies tend to focus on the isolates recovered from one or a few hospitals in one city (sometimes a few cities) from a given country. Clearly, these studies have been very important in understanding the clones spreading in individual countries. However, global genomic epidemiology studies are still a rare occurrence. Studies considering different countries from different continents are clearly lacking. In this regard, the study by Müller and colleagues (10) recently published in mBio is a step forward. Müller et al. (10) sequenced 313 CRAB isolates from 114 hospitals in 47 countries from Africa, Asia, Europe Latin America, and North America. The authors used these data to provide one of the most contemporary panoramas of the distribution of the major International Clones (ICs) and that of Carbapenemase genes as well. To determine the molecular epidemiology of the data set, the authors used an array of traditional (the classic 7-loci MLST Oxford and Pasteur schemes and the bla_OXA-51-like_ marker) and state-of-the-art (core genome MLST and a phylogeny based on whole-genome Single Nucleotide Polymorphisms) genotyping strategies. Whereas for the Carbapenemase genes, they employ an *in silico* prediction, using ResFinder, and a multiplex-PCR. The authors noted that the vast majority of the isolates (92.3%) could be assigned to the well-known ICs; isolates were ascribed from IC1 to IC8. They also found that IC2 had the most extensive distribution and was the largest IC with 62.6% of the isolates; this clone is known to have spread in many parts of the world (11). The second most frequent clone was IC5 (with 14.1%), a lineage described as endemic in Latin America (12). Importantly, given the diversity of the isolates considered, they found two novel clades (clones) well differentiated from the established ICs (IC1 to IC8). One was described as a new IC, namely IC9, and was formed by isolates collected from South Asia (Pakistan), Northeastern Africa (Egypt), and Europe (Belgium and Italy). The other clade was designated USA-clone-1 as the isolates came from four US states. Of note, for IC1, IC2, IC5, IC7, and IC9, the authors also included publicly available genomes for a finer phylogenetic characterization. As for the Carbapenemases, most isolates (over 95%) had the acquired Carbapenemases OXA-23-like (74.8% of isolates) and OXA-40-like (17.9% of the isolates). Around 4% of the isolates did not have an acquired Carbapenemase gene but had an intrinsic OXA-51-like Carbapenemase with an ISAba1 upstream. Finally, just seven isolates (2.2%) had B metallo-beta-lactamase genes (bla_NDM-1_ and bla_IMP-26_). Overall, this study gives a global overview of the geographic distribution of the ICs and the Carbapenemase genes. This study is a solid update on a seminal (pre-genomic) molecular epidemiology study, published in late 2009, that showed the global dissemination of CRAB and recognized the now well-established IC1 to IC8 (11).

Although there are a few recent genomic epidemiology studies (13, 14) that have used considerably many more genomes than Müller et al. (10), these studies conducted convenience sampling—they used published genomes from many different studies—and, thus, the isolates were not sampled using the same criteria and came from different decades. In terms of the sampling, and improving on previous studies, Müller et al. (10) tried to correct for the population size of the countries considered when choosing the isolates to analyse. They also limited the impact of many isolates coming from local outbreaks by restricting the number of isolates to one isolate per hospital per year. However, an important sampling bias by Müller et al. (10) is that they just sampled meropenem-resistant *A. baumannii isolates*. This clearly excluded all the isolates that were susceptible to meropenem. The other sampling issue is that sampling covers just 4 years (isolates were collected between 2012 and 2016). Last but not least, many regions/countries were not included in this study; for instance, most of Africa. Some of these issues are without doubt due to practical reasons, for instance, the lack of partners in many countries/regions of the world or the lack of capacity of the hospitals involved in the sampling to collect both susceptible and antibiotic-resistant isolates. Thus, these types of issues are to be expected. Notwithstanding these issues, the study by Müller et al. (10) is one of the most extensive studies in terms of geography and also considering the number of ICs.

There are several ways in which future genomic epidemiology studies can be enhanced in terms of sampling. First, we should go beyond just focusing on carbapenem-resistant human isolates. Ideally, we want to consider all types of isolates regardless of their antibiotic resistance profile. Second, most of the genomic diversity of this species has been described considering human clinical isolates. However, in the last decade, several studies have clearly shown that *A. baumannii* can dwell in different non-human sources from diverse types of animals to plants (15–19). It has even been suggested that *A. baumannii* must be considered a One Health issue (20, 21). Along these lines, the non-human populations of *A. baumannii* could have important human public health implications, in terms of their pathogenic and antibiotic resistance potential (22). Thus, if we are to get a better grasp of the genomic epidemiology of this species, the next step will be to include not only human but also non-human isolates in our global sampling efforts. In my view, we should be aiming for a global, multi-host genomic epidemiology of this bacterium if we are to fully appreciate the transmission dynamics, antibiotic resistance, and virulence potential of this species.
